# Evidence for sex difference in the CSF/plasma albumin ratio in ~20 000 patients and 335 healthy volunteers

**DOI:** 10.1111/jcmm.13767

**Published:** 2018-07-27

**Authors:** Cristina Parrado‐Fernández, Kaj Blennow, Magnus Hansson, Valerio Leoni, Angel Cedazo‐Minguez, Ingemar Björkhem

**Affiliations:** ^1^ Division of Neurogeriatrics Center for Alzheimer Research Department of Neurobiology, Care Sciences, and Society Karolinska Institutet Huddinge Sweden; ^2^ Clinical Neurochemistry Laboratory Institute of Neuroscience and Physiology Sahlgrenska, University Hospital Mölndal Sweden; ^3^ Division of Clinical Chemistry Department of Laboratory Medicine Karolinska University Hospital Huddinge Sweden; ^4^ Laboratory of Clinical Chemistry Hospital of Varese ASST‐Settelaghi Varese Italy

**Keywords:** brain‐barriers integrity, CSF/plasma albumin ratio, sex differences

## Abstract

Given sex‐related differences in brain disorders, it is of interest to study if there is a sex difference in the permeability of the blood‐cerebrospinal fluid barrier (BCSFB) and the blood‐brain barrier (BBB). The CSF/serum albumin ratio (Q_A__lb_) is a standardized biomarker that evaluates the function of these barriers. In previous studies, contradictory results have been reported with respect to sex difference using this quotient, possibly because of small population sizes and heterogeneity with respect to ages. Q_A__lb_ measurements in more than 20 000 patients between 1 and 90 years visiting our hospitals revealed a significant sex difference in all age groups also when excluding patients with pathologically high CSF albumin > 400 mg/L. Similar pattern was found in 335 healthy volunteers in similar age intervals. Although also other factors are likely important, our observation is consistent with lower integrity of the brain barriers in males. If the difference in Q_A__lb_ is caused mainly by a difference in barrier function, this may require different drug doses and strategies for efficient central nervous system (CNS) delivery in males and females, as well as it may indicate differences in brain metabolism. Moreover, our study emphasizes that different reference values should be used both for different ages and sexes.

## 1 INTRODUCTION

The BBB and the BCSFB selectively regulate the transfer of molecules between the blood, brain parenchyma and CSF^.^ Both blood‐CNS barriers are affected during brain ageing possibly preceding neuronal degeneration.^1^

The Q_Alb_ is a standardized biomarker reflecting the function of these barriers.^2^ As albumin is almost exclusively produced in the liver, increased Q_Alb_ indicates brain damage.^3^ However, reduction in CSF drainage or production, and low turnover rate may also account for increased CSF albumin levels.^2^, ^5^, ^6^, ^7^

Given the sex‐related difference in prevalence and incidence of brain disorders^8^ as well as in drug absorption, bioavailability and its response in brain,^9^ we have explored an aspect rarely investigated on: whether or not there is a sex difference in the permeability of the brain barriers.

In the past, we measured Q_Alb_ in controls and patients with a broad spectrum of neurological diseases.^10^ When re‐analysing our data by grouping the subjects into sex category, we found a significant sex‐related difference in all the populations studied, including the control subjects (Leoni et al, unpublished). Moreover, male patients with lower lumbar pain without positive findings in myelography were reported to have higher CSF albumin than corresponding female patients.^11^ A similar sex difference was found in a population of AD patients.^12^

Contrary to the above studies, no significant sex difference was reported in the Q_Alb_ of 93 subjects searching advice at a neurological clinic without showing any pathological findings.^13^ A later study on 105 healthy volunteers also failed to find a sex difference.^6^ A weakness in all the studies referred to above is the relative small population size. Age heterogeneity might mask sex differences in terms of Q_Alb_. Indeed, most of the studies showing sex difference present a narrow age distribution.

Based on Q_Alb_ measurements on a great number of patients (>20 000) in our hospitals during 8 years and healthy volunteers (n = 335) with a range of age between 1 and 90 years, evidence is presented here for a sex difference in Q_Alb_.

## 2 METHODS

Results for CSF and plasma albumin of 27 263 measurements anonymous patients were obtained in routine diagnostic procedures from the clinical chemistry laboratory at the Karolinska University Hospital Huddinge and Solna during 2008‐2016. Quantification of CSF albumin was performed using immunochemical assays from either Siemens or Roche Diagnostics, and plasma albumin was quantified using either the Siemens immunochemical assay or a bromocresol purple assay from Roche Diagnostics or Beckman Coulter. For multiple measurements on a sample, the mean value was calculated and used in subsequent calculation. In some cases, more than one measurement on the same individual may be present, because of the anonymity of the samples. We estimate that the number of unique patients to be over approximately 20 000.

Data from the previous study on 105 healthy volunteers^6^ were expanded with additional 230 healthy volunteers and grouped into sex categories. The studies were approved by the local ethical committee.

For statistical computing and graphics, we used GraphPad Prism Software, version 5.0. Q_Alb_ did not show a normal Gaussian distribution. Non‐parametric group comparisons of means were performed using Kruskal‐Wallis test followed by Dunn's post hoc test. Two‐tailed *P*‐values < 0.05 were considered as statistically significant.

## 3 RESULTS

**Figure 1:**
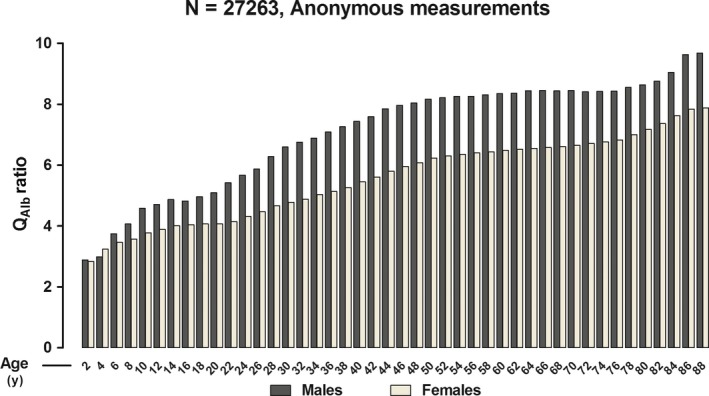
Anonymous Measurements of the Cerebrospinal Fluid/Plasma Albumin Ratio (Q_A__lb_ Ratio) in Males and Females between 1 and 90 y. Data (N = 27 263) are displayed as median and are grouped in 2‐y age interval

Figure 1 shows the medians of Q_Alb_ values obtained from 27 263 measurements on at least 20 000 anonymous patients grouped by age range. No marked sex differences were observed in childhood or at puberty or menopause. We found greater age‐dependent changes than those related to sex. Differences in Q_Alb_ were because of changes in CSF albumin levels rather than plasma albumin levels. The overall sex difference of the latter levels was only 1.5%.

**Figure 2:**
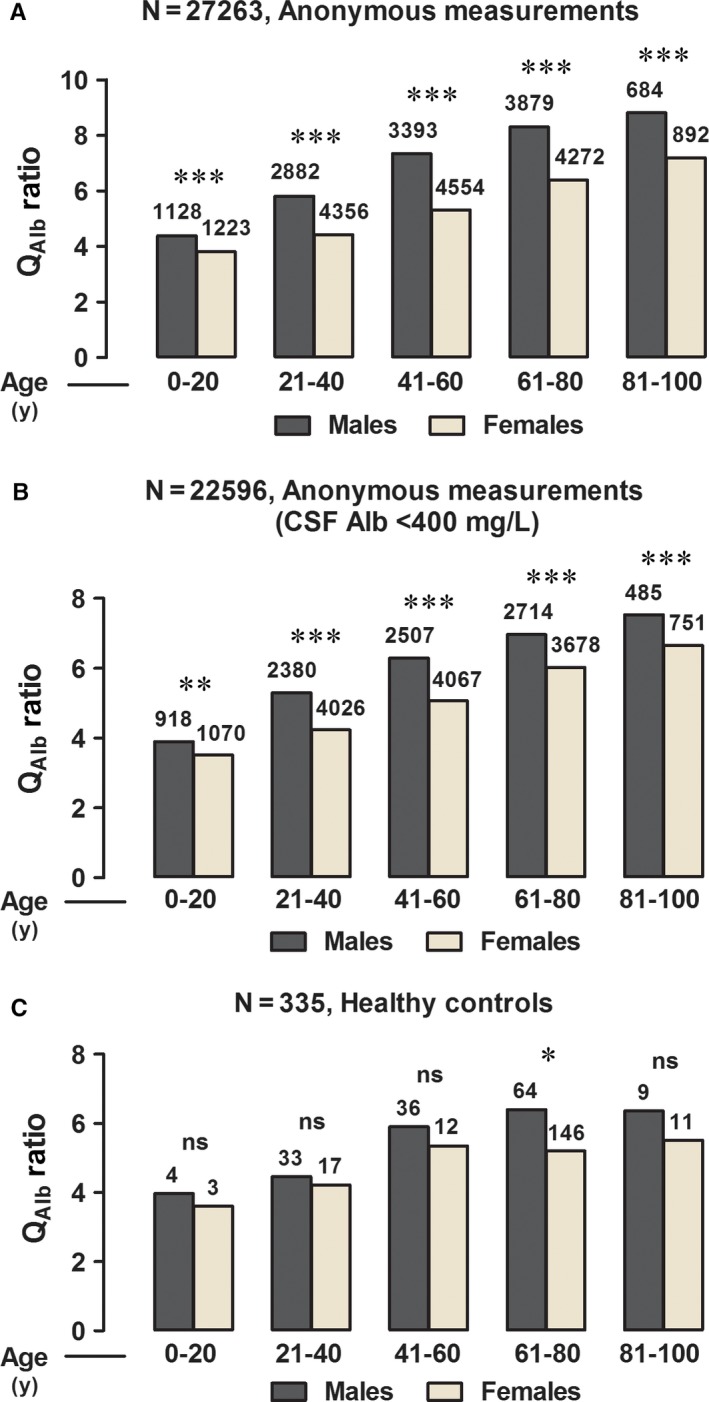
Measurements of Q_A__lb_ Ratio from Anonymous Patients before (A, N = 27 263) and after (B, N = 22 596) Removing CSF Albumin Levels (>400 mg/L); and Q_A__lb_ Ratio from Healthy Controls (C, N = 335). Data are expressed as the median of males and females grouped in 20‐y interval. Kruskal‐Wallis test followed by Dunn's multiple post hoc test (Two‐tailed *P < 0.05, **P < 0.01, ***P < 0.001)

Figure 2A shows the same data grouped in 5 different age intervals. Figure 2B shows the median values after removing the patients with pathologically high CSF albumin levels (>400 mg/L). The sex difference was statistically significant in all age groups. Figure 2C shows in same age intervals, the median Q_Alb_ values from the previous investigation on 105 healthy volunteers^6^ expanded with additional 230 healthy subjects. The sex difference was statistically significant in the age interval 61‐80 but not in the other age categories, probably because of the lower number of subjects. However, Q_Alb_ in the different age groups was found to be very similar to that of the anonymous patients after truncation (Figure 2B).

## 4 DISCUSSION

In the present study Q_Alb_ was measured in more than 20 000 anonymous subjects. This quotient is often included in the investigation of patients searching for headache or suspected neurological or neurodegenerative diseases. The number of measurements was about 25% higher for females.

Here, we uncover a sex difference in Q_Alb_ evident from around 6 years of age up to 90 consistent with a greater integrity of the BCSFB and/or BBB in females. If the observed sex‐related difference in Q_Alb_ is caused only by a difference in barrier function, this may require different drug strategies for efficient CNS delivery in males and females.

Many studies have previously defined sex‐based effects on CNS disease and progression, and here we add an additional sex‐dependent factor that might be relevant. BBB dysfunction has been suggested to be causative for many CNS diseases; however, sex differences have not been discussed in this connection.

The fact that the sex difference was not markedly changed at puberty or at menopause in our study population does not support the contention that hormonal factors are of major importance. The possibility must be considered that sex chromosome genes are more important for the differences observed than hormonal levels.^8^

A limitation of the study is the unavailability of data related to bodyweight. Part of the sex differences may be explained by the higher body mass in males. Seyfert, et al^7^ demonstrated that body mass index (BMI) influences on Q_Alb_. Moreover, high BMI at middle age can predict high Q_Alb_ levels at an advanced age. In this context, we cannot exclude other determinants that may influence to increased CSF albumin levels such as reduced CSF flow‐rate, especially in elderly patients.

If the sex difference is because of a higher proportion of male patients with diseases associated with BBB disturbance, this difference should be reduced after removal of the patients with the highest ratios. After truncation, there was a decrease in the sex difference. A general problem when evaluating levels of different factors in CSF is the generation of suitable reference values. A common strategy is to analyse CSF from patients who have been searched the neurological clinic for subjective symptoms with no objective findings. Healthy volunteers represent the most optimal reference population, but it is difficult to get such subjects motivated to donate CSF. Here we were able to recruit 335 healthy subjects for our study. The age distribution was not optimal with most of the subjects in the age category 61‐89 years, which provided significant sex difference. The pattern was very similar in all age intervals to that obtained in the population of anonymous patients.

Regardless of the relative importance of the different factors that may affect Q_Alb_ our study emphasizes that different reference values should be used both as for different ages as for the two sexes.

## POTENTIAL CONFLICTS OF INTEREST

The authors declare no conflict of interest.

## AUTHOR CONTRIBUTION

CPF, KB, MH, ACM and IB conceived and designed the study. MH, KB and IB contribute to acquisition of data. CPF, KB, MH, VL and IB contribute to analysis of data and/or to drafting the text and/or preparing the figures.
